# Retraction: HNF-4α inhibits hepatocellular carcinoma cell proliferation through mir-122-adam17 pathway

**DOI:** 10.1371/journal.pone.0283891

**Published:** 2023-03-29

**Authors:** The PLOS ONE Editors

After this article [1] was published, the authors requested retraction and notified the journal that they had identified an error in the results: the HNF4α-AS1 gene (a complementary antisense RNA of HNF-4α gene) was mistaken for the HNF-4α gene in the bioinformatic analysis of the GSE84402 dataset.

Disregarding the erroneous bioinformatic analysis results about HNF-4α expression in the GSE84402 dataset, the article’s main conclusion relies on data obtained in cell line experiments. There are concerns about the validity of the control cell line used in comparisons of HCC cell line vs. control cell line expression: as is noted in the Discussion, the control cell line used is of embryonic hepatic origin and as such may not serve as an appropriate control for experiments using adult HCC cell lines. Furthermore, the study did not include loss of function studies or studies in native tissue samples to support claims about a role of HNF-4α in HCC proliferation.

The *PLOS ONE* Editors concluded that the article’s conclusions are not adequately supported when disregarding the erroneous bioinformatic analysis results and considering the methodological concerns discussed above. Therefore, the *PLOS ONE* Editors retract this article.

All authors did not comment on the retraction decision.
